# Interpersonal Fairness, Willingness-to-Stay and Organisation-Based Self-Esteem: The Mediating Role of Affective Commitment

**DOI:** 10.3389/fpsyg.2019.01315

**Published:** 2019-06-11

**Authors:** Samuel Doku Tetteh, Joseph Osafo, Michael Ansah-Nyarko, Kwesi Amponsah-Tawiah

**Affiliations:** ^1^Department of Psychology, University of Ghana, Accra, Ghana; ^2^Department of Organisation and Human Resource Management, University of Ghana Business School, Accra, Ghana

**Keywords:** interpersonal fairness, affective commitment, organisation-based self-esteem, willingness-to-stay, manufacturing industry

## Abstract

This study examines the direct and indirect effects of interpersonal fairness on employees’ willingness-to-stay and organisation-based self-esteem through affective commitment among manufacturing workers in Tema, Ghana. Using the survey design, 300 manufacturing workers in Tema were conveniently sampled for the study. The confirmatory factor analysis and structural equation modelling were used to analyse the data. Results indicated that affective commitment partially mediated the relationship between interpersonal fairness and employees’ willingness-to-stay. Affective commitment also fully mediated the interpersonal fairness-organisation-based self-esteem relationship. Results further showed that organisation-based self-esteem partially mediated the affective commitment and willingness-to-stay relationship, such that, an increase in organisation-based self-esteem leads to a decrease in employees’ willingness-to-stay. These findings emphasised the roles of interpersonal fairness and affective commitment in organisations, where affective commitment increases as a result of an increase in interpersonal fairness and makes employees have an intention-to-stay. The findings imply that employees who are very confident and have higher organisation-based self-esteem stand lower chances of staying in their current organisations. This study is the first to examine how affective commitment transfers the effects of interpersonal fairness unto employees’ intention-to-stay among manufacturing workers in Ghana.

## Introduction

Employee retention has become a crucial issue of concern in present-day organisations due to the negative influence that high rate of turnover has on organisations: skilled labour shortage, high employee turnover and fluctuating economic growth (Aslam et al., 2011).

Andrew Carnegie, cited in Aguenza and Som (2012), remarked, ‘Take away my factories, my plants; take away my railroads, my ships, my transportation, take away my money; strip me of all of these but leave me my key employees, and in two or three years, I will have them all again’. Employee loyalty (willingness-to-stay) has decreased over the past 5 years (Prabhakar, 2016). Loyalty from customers, increased performance, satisfied co-workers and effective succession planning are some of the benefits derived from employee retention (Das and Baruah, 2013). Meanwhile, the adverse effects of employees leaving their organisations are enormous: huge financial costs, interference of co-workers’ relations, additional work stress, low quality of work and diminished abilities to adapt to uncertain environments (Prabhakar, 2016). Despite these adverse effects of turnover, it can also be beneficial when low-productivity workers are more likely to leave (i.e., functional turnover): Such an argument implies that optimal turnover rate is not zero (Madariaga et al., 2018). This led Lee (2018) to empirically conclude that elimination of poorly performing employees contributes to improve organisational performance.

Employees’ willingness-to-stay has been studied with different terminologies such as turnover intentions, employee retention, intention-to-stay/leave, etc. In this study, the term is synonymous to continuance commitment, which refers to the willingness of employees to remain in their current organisations given the option of leaving. Despite the numerous benefits of retention, researchers have largely concentrated on turnover (and its intention) instead of retention (Cardy and Lengnick-Hall, 2011). Mitchell et al. (2001) argued that turnover and retention are different constructs and hence should not be considered as two sides of the same coin. This is because, determinants of employees’ intention-to-leave are different from the antecedents of intention-to-stay. For example, according to Roodt (2018), workload, contract breach, work-family conflict and job insecurity are responsible for employees’ intention-to-leave. However, antecedents of employees’ intention-to-stay include perceived organisational support, quality of supervisors’ relationship, advancement and growth opportunities (Roodt, 2018). According to Yalabik et al. (2017), multiple foci commitment is related to employees’ intention to quit. Yalabik et al. (2017) reported in their study ‘organisational commitment and team commitment are negatively related to intention to quit, whereas, professional commitment is positively related to the professionals’ intention to quit’ (p. 15).

Affective commitment (AC) refers to employees’ emotional attachment to organisations. Meyer et al. (1997) came out with three components, which appear independent of each other. These components are affective commitment, continuance commitment and normative commitment. Affective commitment refers to the employee’s emotional attachment to and immersion in the organisation’s affairs (Meyer et al., 1997). Solinger et al. (2008) criticised the three-component model (TCM) by arguing that ‘general organisational commitment can best be understood as an attitude regarding the organisation, while normative and continuance commitment are attitudes regarding specific forms of behaviour (i.e., staying or leaving)’. They added that TCM does not qualify as ‘a general model of organisational commitment but instead represents a specific model for predicting turnover’ (p. 70). Klein et al. (2012b) also concluded that commitment should be conceptualised as a specific bond denoting a voluntary allegiance and obligation for a particular target. Based on the reconceptualisation of the three-component model by Klein et al. (2012a,b, 2014) proposed a four-item measure for commitment, which reflects affective commitment proposed by Meyer et al. (1997). To conclude, while some studies view affective commitment as the true commitment with all other forms of commitment directed towards some aspect of the workplace (Solinger et al., 2008; Klein et al., 2012a,b, 2014), other studies still adhere to the three-component model. Following this debate, however, no study has tried to test whether the components of the TCM are distinct or very much related, hence belonging to the same construct. The present study, therefore, aims at contributing to the debate by examining the factor structure of affective and continuance commitment (i.e., intention-to-stay or leave) to see if they belong to the same construct or are distinct. According to Klein et al. (2012a,b), the TCM represents a specific model for predicting turnover rather than a general model for commitment. This study examined the role that interpersonal fairness plays in explaining employees’ willingness-to-stay in their current organisations through organisational commitment (i.e., affective commitment) based on the assumption of the affective events theory. It also sought to find out if affective commitment of employees mediates the relationship between interpersonal fairness and their work-related self-esteem. Commitment has significant effects on certain work attitudes and behaviours such as organisational citizenship behaviours (Saraih et al., 2017), counterproductive work behaviours and turnover intentions (Ghandi et al., 2017).

One personal attribute that certainly and consistently enhances the understanding of organisational behaviour is employees’ ‘self-esteem’ on the job (Pierce and Gardner, 2004). It has been found that workers with very high organisation-based self-esteem (OBSE) are less likely to leave their working organisation (Lapointe et al., 2011). Organisation-based self-esteem is defined as the degree to which an individual believes himself or herself to be capable, significant and worthy as an organisational member (Pierce et al., 1989). It has to do with employees’ view of their worth or significance associated with their organisation. It is demonstrated in the self-perceived value that individuals have of themselves as important, competent and capable within their employing organisations. According to Pierce and Gardner (2004), workplace self-esteem is generally determined by an individual’s work and organisational experiences. It seems reasonable to expect that individuals who perceive fairness will also see themselves as valuable to the organisation and hence will be willing to stay within the organisation. Organisation-based self-esteem has a positive relationship with job commitment (Pierce and Gardner, 2004). A meta-analysis by Bowling et al. (2010) established a relation between OBSE and satisfaction, commitment, intention-to-leave, employee health, job performance and organisational citizenship behaviour.

To address the issue of employees leaving their organisations, organisational fairness is one of the main factors that is relevant due to its close relationship with employees’ willingness-to-stay within an organisation (Piccoli et al., 2011). No social organisation will exist without fairness. Fair treatment of workers fosters collaborations and togetherness that facilitates effectiveness and efficiency. Interpersonal fairness refers to equity in relationships that exists among workers as well as their relationship with their supervisors at the workplace. Interpersonal fairness is the centre of attention of all humanistic affairs because people are respond to how they are treated. According to Chegini (2009), when fairness exists, all the works are done correctly, but people have to get their rights illegally if partiality is the order of the day.

Interpersonal fairness concentrates on individuals’ perceptions of the quality of interactive treatment received during the executing of organisational procedures (Jawahar, 2002). This dimension of justice focuses on the fairness the individual receives from the decision-makers (Ambrose et al., 2002). Fairness from both supervisors and co-workers was negatively related to job distress and aggressive behaviours whereas employees were said to respond with dissatisfaction (e.g., intention-to-leave) to unfair treatments from their supervisors (Ladebo et al., 2008). In another study, Raymond et al. (2006) also found a positive direct relationship between perception of justice and job commitment of employees. Arti et al. (2009) also pointed out that fairness in the disbursement of organisation’s resources significantly relates to job satisfaction.

### Theoretical Foundation and Hypotheses

The Affective Events Theory (AET), developed by Weiss and Cropanzano (1996), later revised by Weiss and Beal (2005) serves as the foundation of this study. The theory argues that workplace events elicit emotional responses in employees, which influence workplace cognition and behaviour. AET emphasises the structure, antecedents and consequences of emotional experiences at work (Weiss and Cropanzano, 1996). A major theme of AET is that events are the primary causes of emotional responses/states (Weiss and Beal, 2005). Thus, what happens to people at work affects their feelings about their job, which in turn influences the behaviour and attitude at the workplace. Weiss and Beal (2005) added that emotion-driven behaviours are decisions and judgments that serve as consequences of being in a specific affective state. For the current study, interpersonal fairness is considered as positive work climate created through events/happenings at the workplace, which influences affective commitment (emotional state) of workers. This state, in turn, affects the employees’ willingness-to-stay and their organisation-based self-esteem.

The AET is supported in this study by the social exchange theory (SET; Blau, 1968; Cropanzano and Mitchell, 2005). SET explains employee behaviours in a bilateral communication between the employee and the organisation (Yildiz et al., 2015). According to the theory, employees will reciprocate the positive treatments from employers with positive behaviours such as commitment (Liu et al., 2010) and organisational citizenship behaviour (Suárez-Mendoza and Zoghbi-Manrique-de-Lara, 2008). Likewise, employees also reciprocate negative treatments with negative behaviours and attitudes such as turnover intention (Liu et al., 2010), dissatisfaction and counterproductive work behaviours (Jawad et al., 2013; Sharkawi et al., 2013). In other words, employees respond to how they are treated by their organisation (Mearns et al., 2010).

### Affective Commitment and Willingness-to-Stay

A comprehensive study by Perreira et al. (2018) explored the relationship between interpersonal justice, affective commitment and turnover intentions and reported that interpersonal justice is positively related to affective commitment, which is also negatively related with turnover intentions. Another study by Satoh et al. (2017) found affective and normative commitments having positive effects on nurses’ intention-to-stay in their profession. Tarigan and Ariani (2015) also established a negative relationship between commitment and intention-to-leave among manufacturing workers. Among employees in the casino industry, affective commitment negatively predicted turnover intentions (Kim et al., 2016). In addition, Tirelli and Goh (2015) also reported in their study that, affective commitment has a negative association with employees’ turnover intentions. A study by Kirk-Brown and Van Dijk (2014) compared the predictive strength of affective commitment on turnover intentions between people with chronic illnesses and healthy individuals and found that the relationship between affective commitment and intention-to-leave was stronger for the healthy population than those with chronic illness. Meanwhile, for both populations, the relationship was negative. Also among hospitality front-line employees, high levels of commitment were found to reduce turnover intention (Kang et al., 2015).

### Affective Commitment and Organisation-Based Self-Esteem

Jussila et al. (2012) explored the sources (antecedents) of affective commitment. Affective commitment was found to be explained by three theoretical constructs namely organisational identification, organisation-based self-esteem and psychological ownership. Lapointe et al. (2011) also explored the association between the three types of commitment and OBSE. Findings revealed that continuance commitment exacerbates employees’ OBSE. Khattak et al. (2014) also examined the relationship between affective commitment and OBSE. They reported that affective commitment has a significant positive association with OBSE. Finally, Chen et al. (2016) reported in their study that OBSE has a significant relationship with nurses’ intention-to-stay in the nursing profession.

### Interpersonal Fairness and Willingness-to-Stay

Perreira et al. (2018) explored the relationship between fairness and intention-to-leave. They reported that interpersonal fairness negatively predicts intention-to-leave. Again, Harris et al. (2018) acknowledged that perception of client-focused justice predicts employee turnover intention. Moreover, Vaamonde et al. (2018) found the dimensions of justice (i.e., distributive, procedural and interpersonal justice) have negative indirect effects on turnover intentions through burnout and job satisfaction. These results indicate that distributive, procedural and interpersonal justice perceptions relate to lower levels of burnout, which in turn promote greater job satisfaction and lower turnover intentions among employees.

### Interpersonal Fairness and Affective Commitment

Ogwuche and Apeiker (2016) explored the impact of interactional fairness on organisational commitment among lecturers. Results of the study indicated a significant influence of interactional justice on organisational commitment among lecturers. Another study by Ha and Ha (2015) found a significant positive association between distributive, procedural, interactional justices and affective commitment. Again, Raza et al. (2013) found fairness to be positively and significantly related to organisational commitment. In addition, they argued that increased commitment leads to increase in productivity. Leow and Khong (2009) also found direct effects of organisational justice dimensions on organisational commitment.

### Affective Commitment as a Mediator

Affective commitment has been found to serve as a good mediator in many workplace relations, especially when it comes to actual turnover or turnover intentions. A recent study by Perreira et al. (2018) examined the mediating role of affective commitment on the relationship between interpersonal justice and turnover intentions of health care professionals. Findings of their study indicated that interpersonal justice is positively related to affective commitment but negatively related to turnover intentions. Interpersonal justice also indirectly predicted turnover intentions through affective commitment such that justice increases affective commitment, which also leads to a reduction in turnover intentions. More so, Yang et al. (2018), stated that affective commitment is a partial mediator of the relationship between work-family conflict and voluntary turnover. Fazio et al. (2017) similarly found that AC partially mediates the negative relationship between turnover intention and perceived support. In addition, Dhar (2015) concluded that affective commitment partially mediates the relationship between access to training and service quality of hotel staff. Wang et al. (2014) also established an indirect effect of career goal progress on employees’ voice behaviour through affective commitment. They concluded that affective commitment mediated the relationships between dimensions of career growth opportunities and voice behaviour of employees. Further, according to Martin-Perez and Martin-Cruz (2015), reward systems indirectly predicted knowledge transfer *via* affective commitment. They argued that AC is necessary to the intensification of employees’ loyalty, lessening turnover intentions, and improve their willingness to transfer their knowledge. In another study by Demirtas and Akdogan (2015), affective commitment was found to mediate the relationship between ethical leadership and turnover intentions. Based on the literature review, the current study tested the following hypotheses.

*Hypothesis 1:* Interpersonal fairness will predict affective commitment of employees.

*Hypothesis 2:* Affective commitment will also predict OBSE.

*Hypothesis 3:* Affective commitment will predict employees’ willingness-to-stay.

*Hypothesis 4:* OBSE will have a significant relationship with willingness-to-stay.

*Hypothesis 5:* Affective commitment will mediate the relationship between interpersonal fairness and willingness-to-stay.

*Hypothesis 6:* Affective commitment will also mediate the fairness-OBSE relationship.

## Materials and Methods

### Participants

The study sampled and tested 300 manufacturing workers in Tema from the Greater Accra region of Ghana. These workers were selected due to the high rate of turnover in the manufacturing industry. According to the National Employment Report (2014), the manufacturing sector is the second highest employer of workers in Ghana after the retail and wholesale sector. The data were collected at two different times from same organisations within a 1-year interval. At time I, the data on the perception of interpersonal fairness, affective commitment and demographic characteristics were collected. Respondents were made to answer the survey questions printed on sheets of paper. These workers were contacted at lunchtime in the respective organisations. Out of 400 questionnaires distributed at time I, 375 were retrieved representing a response rate of 93.8%. At time II, 375 questionnaires were distributed, of which 330 returned (response rate of 88%). For the second round, respondents answered questions relating to their willingness-to-stay and organisation-based self-esteem. Finally, 300 questionnaires were found useful for analysis after reconciling the two responses and taking away those questionnaires that had a chunk of missing data.

Out of the 300 participants used for this study, 152 (representing 50.7%) were males while the remaining (148, 49.3%) were females. In addition, more than 50% (57.7%) of the sample were at most 30 years of age. This shows that the participants for the study were relatively young.

In terms of tenure, 80% of the respondents have been working between 1 and 10 years. Only 7.7% have been with their organisations for more than 15 years. More so, almost 50% (48.3%) of the sample had secondary school education, while 35.3% were graduates.

### Measures

Interpersonal fairness was measured using four items found on the interactional justice scale of Colquitt’s (2001). The items were measured on a five-point Likert scale (1–strongly disagree to 5–strongly agree) with a Cronbach’s alpha of 0.89. A sample item is ‘Have they treated you with respect?’

Affective commitment was also assessed with eight items from Meyer and Allen’s (1991) commitment scale on a five-point Likert scale (1–strongly disagree to 5–strongly agree) with a reliability coefficient of 0.87. A sample item is ‘I feel a strong sense of belonging to this organisation’.

Meyer and Allen’s (1991) continuance commitment was used to assess employees’ willingness-to-stay. It comprised seven items, which were measured on a five-point Likert scale with a Cronbach’s alpha of 0.81. ‘I would be very happy to spend the rest of my career with this organisation’ is a sample item.

The English version of Pierce’s organisation-based self-esteem scale (Pierce et al., 1989) was used to measure OBSE. The scale comprised 10 items, with a Cronbach’s alpha of 0.83. A sample item is ‘I am valuable in this organisation’.

## Results

The confirmatory factor analysis (CFA) and structural equation modelling (SEM) were used to evaluate the measurement model and to test the study hypotheses respectively. Considering the number of items per scale, parcels were created per latent variable to retain a higher quotient of the indicator to sample size (Landis et al., 2000). After establishing the parameters of the goodness of fit indices for the measurement model of all the four variables, the SEM was used to test the study hypotheses. Before testing the hypotheses, descriptive statistics and normality of the data were verified. Results are presented below.

### Descriptive Statistics

In Table 1, it was observed that the data had no skewness and kurtosis problems; all variables were within the acceptable range of ±1. In addition, mean and standard deviation were examined. The means (standard deviations) of the study variables were: affective commitment 24.59 (±6.142), willingness-to-stay 28.67 (±7.157), organisation-based self-esteem 28.97 (±4.737) and interpersonal fairness 14.23 (±3.893). It is important to add at this point that all the demographic variables have been eliminated from the analyses as potential controls since they did not have any significant effect on any of the two dependent variables considered in this study based on the recommendations of Becker (2005) and Carlson and Wu (2012).

**Table 1:** Descriptive statistics and normality.

Variables	Mean	Standard deviation	Skewness	Kurtosis
Affective commitment	24.59	6.142	−0.184	−0.509
Willingness-to-stay	28.67	7.157	−0.102	−0.306
Organisation-based self-esteem	28.97	4.737	−0.707	−0.015
Interpersonal fairness	14.23	3.893	−0.213	−0.645

### Measurement Model

#### Goodness of Fit Indices

**Figure 1:**
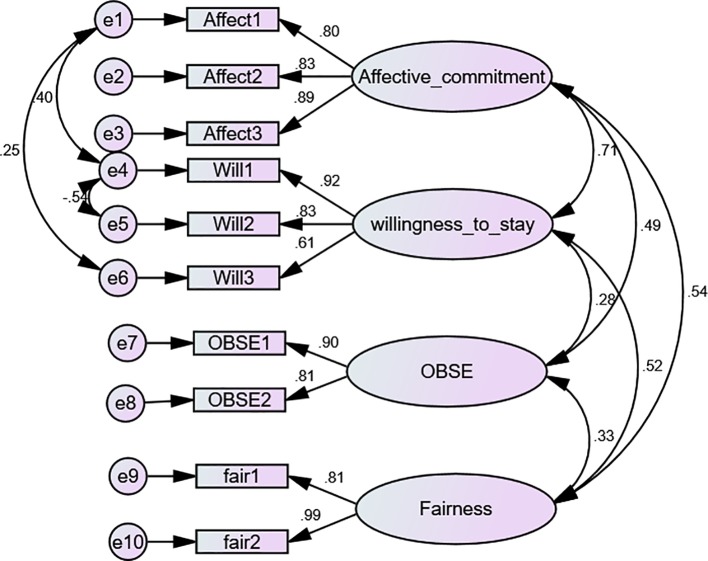
CFA model showing the factor loadings and covariance among the study variables.

The measurement model assessed the correlation between the parcelled items and the various study constructs. This was done in order to find out how good these items were in predicting the latent variables (Byrne, 2010). Confirmatory factor analysis (see Figure 1) was used to test the four-factor measurement model of affective commitment, willingness-to-stay, organisation-based self-esteem and interpersonal fairness. To evaluate the model fitness, the *χ*^2^ goodness of fit test (CMIN), standardised root mean squared residual (SRMR), root mean square error of approximation (RMSEA), Tucker and Lewis index (TLI), goodness of fit index (GFI) and the comparative fit index (CFI) were examined. The measurement model produced an excellent fit for the data with *χ*^2^ (26) = 47.499, *χ* ^2^/df = 1.827, SRMR = 0.030, RMSEA = 0.053, TLI = 0.978, GFI = 0.971, (CFI) = 0.987, 90% CI (0.028, 0.076), PClose = 0.400 (Hu and Bentler, 1999).

#### Convergent and Discriminant Validity

Construct (convergent and discriminant) validity was examined using factor loadings, composite reliability (CR) and average variance extracted (AVE; Hair et al., 2010). As demonstrated in Table 2, there were no concerns in terms of convergent validity because the standardised coefficients from items to factors ranged from 0.638 to 0.818, with statistically significant regression weight, and all items exceeded the conventional cut-off of 0.50 (Hu and Bentler, 1999). The composite reliability values that depict the level at which the factors (items) specify the unobserved variables ranged from 0.837 to 0.899, which are greater than the criterion value of 0.7 (Hair et al., 2010). More so, discriminant validity was assessed using the maximum-shared variance (MSV), construct correlations and a square root of AVE. The result showed that the MSV ranges from 0.238 to 0.498. According to Hu and Bentler (1999), an adequate discriminant validity is achieved when the scores of the MSV are lower than the AVE. In addition, the construct correlations are lower than the square root of AVE. Observing from Table 2, MSV scores were lower than AVE scores and the square root of AVE scores was higher than the construct correlations showing adequate discriminant validity.

**Table 2:** Reliability, validity, goodness of fit indices and inter-correlations among the study variables in the model.

Variables	*α*	CR	MaxR	AVE	MSV	AC	WS	OBSE	Fairness
AC	0.865	0.879	0.887	0.708	0.498	**(0.842)**			
WS	0.810	0.837	0.893	0.638	0.498	0.705	**(0.799)**		
OBSE	0.825	0.845	0.862	0.732	0.238	0.488	0.278	**(0.856)**	
Fairness	0.890	0.899	0.974	0.818	0.289	0.538	0.517	0.333	**(0.904)**

CMIN = 47.499, DF = 26, CMIN/DF = 1.827, CFI = 0.987, SRMR = 0.030, RMSEA = 0.053, 90% CI (0.028, 0.076), PClose = 0.400, *N* = 300.

Note: CR, Composite reliability; AVE, average variance extracted; MSV, maximum shared variance; MaxR, maximum reliability; AC, affective commitment; WS, willingness-to-stay; OBSE, organisation-based self-esteem; square root AVE (in bold parentheses).

#### Reliability (Internal Consistency)

Reliability was also assessed using Cronbach’s alpha. In Table 2, affective commitment had *α* = 0.865, willingness-to-stay *α* = 0.810, organisation-based self-esteem *α* = 0.825 and interpersonal fairness *α* = 0.890. All alpha values exceeded the accepted value of 0.7, signifying high levels of reliability (Hair et al., 2010).

### Structural/Hypothesised Model

#### Structural Model Indices

**Figure 2:**
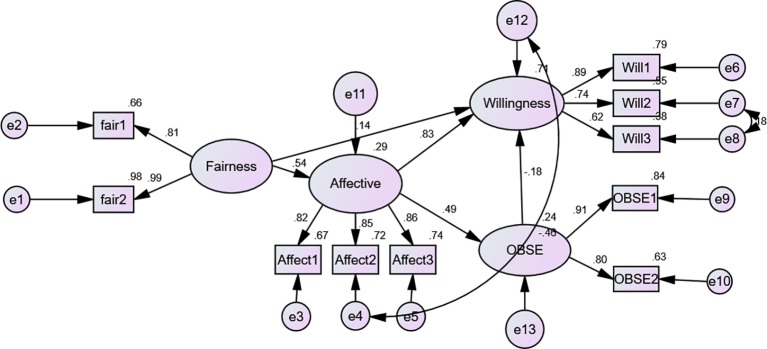
The structural model.

Results obtained from the structural model test (see Figure 2) showed very good fit indices from the study data, with χ^2^/df = 1.725, GFI = 0.969, CFI = 0.988, TLI = 0.981, SRMR = 0.037, RMSEA = 0.049, 90% CI (0.024, 0.072) and PClose = 0.492. Preceding the analysis, all variables used in the models were tested for multicollinearity, using the results of variance inflation factor (VIF). All VIF values obtained were less than 2, which is far less than the accepted value of 10 (Hair et al., 2010) indicating no issues with multicollinearity.

### Hypotheses Testing

The results of the regression model showed coefficient of determination (*R^2^*) values ranged from 9.8 to 42.6% of the variance, which signified a weak to moderate amount of variance explained. In addition, the results indicated that interpersonal fairness has a significant effect on affective commitment (*β* = 0.54, *p* < 0.001). Similarly, affective commitment is found to have a positive influence on organisation-based self-esteem (*β* = 0.49, *p* < 0.001), lending support to both H1 and H2. In addition, affective commitment had a significant effect on employees’ willingness-to-stay (*β* = 0.83, *p* < 0.001). Likewise, organisation-based self-esteem predicted employees’ willingness-to-stay (*β* = −0.18, *p* < 0.01), offering support for H3 but not H4. Interpersonal fairness also directly predicted employees’ willingness-to-stay (*β* = 0.14, *p* < 0.05).

### Standardised Indirect Effects

The result presented in Table 3 shows that the standardised indirect (mediated) effect of interpersonal fairness on willingness-to-stay is 0.403, *p* = 0.001. That is, due to the indirect effect of interpersonal fairness on willingness-to-stay, when interpersonal fairness increases by 1 standard deviation, willingness-to-stay also increases by 0.403 standard deviations. However, the direct effect of interpersonal fairness on willingness was significant, suggesting a partially mediated relationship by affective commitment; hence, H5 was supported. In addition, the standardised indirect (mediated) effect of interpersonal fairness on organisation-based self-esteem is 0.264, *p* = 0.001. That is, due to the indirect effect of interpersonal fairness on organisation-based self-esteem, when interpersonal fairness increases by 1 standard deviation, OBSE also increases by 0.264 standard deviations in addition to any direct effect that fairness may have on OBSE. There was no significant direct influence of fairness on OBSE after inclusion of the mediated variable (affective commitment) showing a full mediated relationship; hence, H6 was supported. More so, the indirect (mediated) effect of affective commitment on willingness-to-stay through OBSE is −0.09, *p* = 0.001, meaning a unit increase in affective commitment leads to 0.09 decreases in employees’ willingness-to-stay adding to any direct effect that affective commitment may have on willingness-to-stay.

**Table 3:** Standardised indirect effects.

Variables	Fairness	AC	OBSE	WTS
AC	–	–	–	–
OBSE	0.264^***^	–	–	–
WTS	0.403^***^	−0.086^***^	–	–

AC, affective commitment; OBSE, organisation-based self-esteem; WTS, willingness-to-stay.

*** p < 0.001.

## Discussion

This study examined the role of interpersonal fairness in predicting employees’ willingness-to-stay directly and indirectly through affective commitment. Results indicated that interpersonal fairness was directly and indirectly related to willingness-to-stay: the indirect effect was mediated by affective commitment. In other words, affective commitment partially mediated the relationship between interpersonal fairness and willingness-to-stay. This finding agrees with the findings of other studies (i.e., Ha and Ha, 2015; Ogwuche and Apeiker, 2016). These studies established a significant positive relationship between interpersonal fairness and commitment. In addition, the mediating role of affective commitment has been established on the relationship between organisational justice and employees’ intention-to-stay (Demirtas and Akdogan, 2015; Fazio et al., 2017; Perreira et al., 2018; Yang et al., 2018). This means that when employees perceive fair treatment at work, they develop emotional attachment to their organisations and the work that they do. This is very important in that, in Ghana and most African countries, interactions at the workplace are very important and hence must be encouraged and channelled in a way that they will enhance the effectiveness of the organisation. The finding is also similar to the finding of other studies (Leow and Khong, 2009; Raza et al., 2013). These studies reported that interpersonal fairness in organisations makes employees develop a sense of identification to their organisation, which makes them have the intention-to-stay with the organisation. Based on these findings, the study recommends that issues of justice and fairness are included in the routine training and seminars for both employees and managers to know how to act fairly at the workplace.

This study also found that affective commitment has a full mediating effect on the relationship between interpersonal fairness and OBSE. Thus, interpersonal fairness makes employees develop affective commitment towards their organisations, which in turn leads to the formation of a positive self-concept on the job (OBSE). This outcome is supported by earlier studies (i.e., Lapointe et al., 2011; Jussila et al., 2012; Khattak et al., 2014). All these studies acknowledged the crucial role of commitment in the realisation of OBSE. For instance, Jussila et al. (2012) established that affective commitment predicts organisation-based self-esteem. The present study also highlights the role that interpersonal fairness plays in today’s organisations that has a greater diversified workforce. Hence, interactional fairness must be encouraged among co-workers and supervisors.

Interestingly, the current study also found that OBSE partially mediates the relationship between affective commitment and willingness-to-stay. Meaning, an increase in OBSE leads to reductions in employees’ willingness-to-stay. Meanwhile, as reported earlier, there was a very strong direct effect of affective commitment on intention-to-stay. This finding would mean that an employee who is affectively committed to the organisation does not need OBSE to develop willingness-to-stay. Moreover, if an employee with AC also develops a very high OBSE, she or he is likely to decrease the willingness-to-stay in the present organisation. Having a high OBSE implies that the employee is confident enough to secure other jobs when she or he leaves the current job. In other words, workers who believe in themselves and what they do would be willing to explore other opportunities elsewhere and will take on new challenging jobs to match their energy levels. It is therefore important that employers identify such employees and give them more roles and responsibilities as well as more autonomy for them to exercise their potentials; otherwise, they are likely to exit.

### Implications of the Study

The findings of this study suggest that interpersonal fairness at the workplace cannot be compromised looking at its beneficial consequences. Management must take keen interest to ensure that there is justice or fairness among workers. Managers and supervisors should be made aware of how their relationships with their subordinates if not fair would be detrimental to the organisation. Leaders should be educated on how to relate fairly to all employees. Training and seminars can be organised for leaders and supervisors on how to exercise fairness and equity in dealing with employees. Interpersonal fairness is manifested in how managers and supervisors relate to employees. It is very crucial for leaders to deal with all their subordinates in the same way without showing any favouritism. Interactions should not be based on any presumed similarity in identification but rather be free and fair. In addition, this study also shows the relevance of affective commitment in organisations. It is expected that, if management exhibits fairness in dealing with employee matters, the latter will become committed to the organisation’s goals, which will lead to positive work behaviours.

In terms of theory and research, the study found that continuance commitment (otherwise termed as willingness-to-stay) and affective commitment are different constructs as suggested by Solinger et al. (2008) and Klein et al. (2012a,b, 2014). Although the two constructs had a high correlation, they had separate factor loadings. Affective commitment should be regarded in future studies as true commitment while continuance and normative commitment be regarded as the commitment to specific acts or behaviours. This study also broadens the research on interpersonal fairness and OBSE by establishing that there is a strong indirect relation between the two constructs through affective commitment. The study is one of the few that test the mediating role of affective commitment and OBSE based on the assumptions of the affective events theory and the social exchange theory.

### Conclusion

The current study examined the direct and indirect effects of interpersonal fairness on employees’ willingness-to-stay and OBSE; the indirect effects were mediated by affective commitment among manufacturing workers in Tema– Greater Accra region, Ghana. The study found a positive significant relationship between interpersonal fairness and affective commitment. Affective commitment was also found to predict employees’ willingness-to-stay and their OBSE. Furthermore, the results indicated that affective commitment partially mediated the relationship between interpersonal fairness and willingness-to-stay; whereas, it fully mediated the interpersonal fairness-OBSE relationship. The study also found significant direct relationships among the variables.

### Limitation and Future Research

The present study in an attempt to lessen the potential effect of common method bias collected the data at two points in time (i.e., Podsakoff et al., 2003). However, this does not imply the study is without any limitation. The current study collected data in only one industry, which has its own characteristics that may differ from other industries. Generalisation of results should be done with caution. This implies that the study could be replicated in other industries to verify if the results will be similar or differ in any way. The study also recommends that future studies explore the relationship between OBSE and intention-to-stay, since it appears the negative relationship found by this study is not in line with earlier studies that found positive relationships (Minibas-Poussard et al., 2017) although self-efficacy was found to have a positive relationship with turnover intentions (Ozyilmaz et al., 2018).

## Ethics Statement

Ethical approval was not necessary and mandatory for the current study regarding the rules and regulations of the Ethics Committee for Humanities’ of the University of Ghana. However, only respondents who were willing participated in the study after a verbal explanation of the purpose of the research was given to them. The researchers sought the approval and consent from the various managers and supervisors of the employees even before contacting the latter. No one was coerced or lured in anyway to be part of the study. All information regarding the study was well presented to the employees and only those who agreed to be part of the study were used.

## Author Contributions

ST and JO conceptualised and designed the study. Data were collected through this collaborative work. MA-N and ST did the data analysis and interpretation. The first draft, which was written by ST, was proofread and edited by KA-T.

### Conflict of Interest Statement

The authors declare that the research was conducted in the absence of any commercial or financial relationships that could be construed as a potential conflict of interest.
